# Zellweger spectrum disorders: clinical overview and management approach

**DOI:** 10.1186/s13023-015-0368-9

**Published:** 2015-12-01

**Authors:** Femke C. C. Klouwer, Kevin Berendse, Sacha Ferdinandusse, Ronald J. A. Wanders, Marc Engelen, Bwee Tien Poll-The

**Affiliations:** Department of Paediatric Neurology, Emma Children’s Hospital, Academic Medical Center, University of Amsterdam, Meibergdreef 9, PO BOX 22660, 1105 AZ Amsterdam, The Netherlands; Laboratory Genetic Metabolic Diseases, Academic Medical Center, University of Amsterdam, Amsterdam, The Netherlands

**Keywords:** Zellweger spectrum disorder, ZSD, Peroxisome biogenesis disorder, PBD, Zellweger syndrome, Neonatal adrenoleukodystrophy, Infantile Refsum disease, Heimler syndrome, *PEX*, Very long chain fatty acids, VLCFA

## Abstract

Zellweger spectrum disorders (ZSDs) represent the major subgroup within the peroxisomal biogenesis disorders caused by defects in *PEX* genes. The Zellweger spectrum is a clinical and biochemical continuum which can roughly be divided into three clinical phenotypes. Patients can present in the neonatal period with severe symptoms or later in life during adolescence or adulthood with only minor features. A defect of functional peroxisomes results in several metabolic abnormalities, which in most cases can be detected in blood and urine. There is currently no curative therapy, but supportive care is available. This review focuses on the management of patients with a ZSD and provides recommendations for supportive therapeutic options for all those involved in the care for ZSD patients.

## Background

The Zellweger spectrum disorders (ZSDs) are a heterogeneous group of autosomal recessive disorders characterized by a defect in peroxisome formation and are caused by mutations in one of 13 *PEX* genes [1–3]. Because of the defect in peroxisome formation, multiple metabolic (both catabolic and anabolic) pathways are impaired resulting in metabolic abnormalities. Typically, ZSD patients accumulate very long chain fatty acids (VLCFAs), phytanic- and pristanic acid, C27-bile acid intermediates and pipecolic acid in plasma and have a deficiency of plasmalogens in erythrocytes [4]. Clinically, ZSDs are highly heterogeneous, but the core features are: liver dysfunction, developmental delay and other neurological abnormalities, adrenocortical dysfunction and hearing- and vision impairment [5]. Before the biochemical and molecular basis of ZSDs was known, they were clinically described as three distinct disorders: Zellweger syndrome (ZS), neonatal adrenoleukodystrophy (NALD) and infantile Refsum disease (IRD). These phenotypes are currently recognized as presentations within a clinical spectrum (with ZS being at the most severe end of the spectrum) which are now collectively referred to as ZSDs, in order to appreciate the wide variations in presentation [6]. Recently, Heimler syndrome was recognized as a peroxisome biogenesis disorder within the Zellweger spectrum and added to the (very) mild end of the clinical spectrum [7]. This review provides a clinical overview of Zellweger spectrum disorders and focuses on management of patients with a ZSD. New developments in the field of management are discussed.

## Disease names and synonyms

Zellweger spectrum disorder/Zellweger syndrome spectrum/Zellweger syndrome/neonatal adrenoleukodystrophy/infantile Refsum disease/Heimler syndrome (ORPHA79189).

## History and definition

Bowen et al. described a syndrome with failure to thrive, congenital glaucoma and craniofacial dysmorphic features with early death (before 2 years of age) [5]. In 1965 Smith et al. described two siblings with comparable multiple congenital malformations, but also polycystic kidneys and intrahepatic biliary dysgenesis [8]. In 1967 Passarge et al. introduced the term cerebro-hepato-renal syndrome. Since Hans Zellweger, a pediatrician, contributed two of the originally described patients it was later called Zellweger syndrome [9]. It was not until 1973 that the causal link between ZS and peroxisomes was made, when Goldfischer et al. described the absence of peroxisomes in hepatocytes and renal proximal tubules [10]. Although the clinical presentation is different, the discovery of similar biochemical abnormalities revealed that the earlier described entities infantile Refsum disease and neonatal adrenoleukodystrophy were also peroxisomal disorders [11, 12]. Based on these findings, peroxisomes which were once considered unimportant organelles, were now connected to a group of diseases and became the object of intensive scientific investigations. It turned out that peroxisomes are important organelles in the eukaryotic cell, and are involved in many catabolic and anabolic metabolic pathways [4, 13]. At present more than 15 different peroxisomal disorders have been identified. The genetic basis of ZSDs has largely been resolved and now includes 13 different *PEX* genes [14, 15]. The group of diseases is now referred to as Zellweger spectrum disorders and include the old disease entities of ZS, NALD, IRD but also Heimler syndrome which was recently recognized as a ZSD [7, 16].

## Epidemiology

The incidence of ZSDs is estimated to be 1 in 50.000 newborns in the United States [17]. It is presumed that ZSDs occur worldwide, but the incidence may differ between regions. For example, the incidence of (classic) Zellweger syndrome in the French-Canadian region of Quebec was estimated to be 1 in 12 [18]. A much lower incidence is reported in Japan, with an estimated incidence of 1 in 500.000 births [19]. More accurate incidence data about ZSDs will become available in the near future, since newborn screening for X-linked adrenoleukodystrophy (X-ALD) will be implemented in several countries [20, 21]. The screening method is based on C26:0-lysophosphatidylcholine (C26:0-lysoPC) measurement in dried bloodspots using LC-MS/MS technology, which will also identify ZSD patients [22].

## Clinical features

**Fig. 1:**
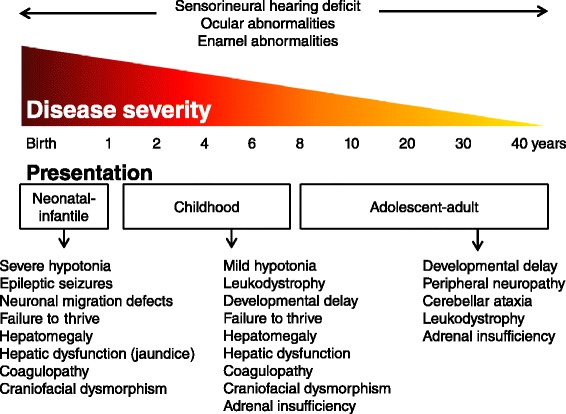
Schematic overview of main presenting symptoms in ZSDs per clinical group

Patients with a ZSD can roughly be divided into three groups according to the age of presentation: the neonatal-infantile presentation, the childhood presentation and an adolescent-adult (late) presentation [23]. An overview of the main presenting symptoms for these groups is summarized in Fig. 1. The original classification of ZS, NALD and IRD is less valuable now, especially since additional variant phenotypes suggestive for a disease spectrum have been identified. For discussing prognosis and counseling patients or families this classification may in some cases still be useful [24].

### Neonatal-infantile presentation

**Fig. 2:**
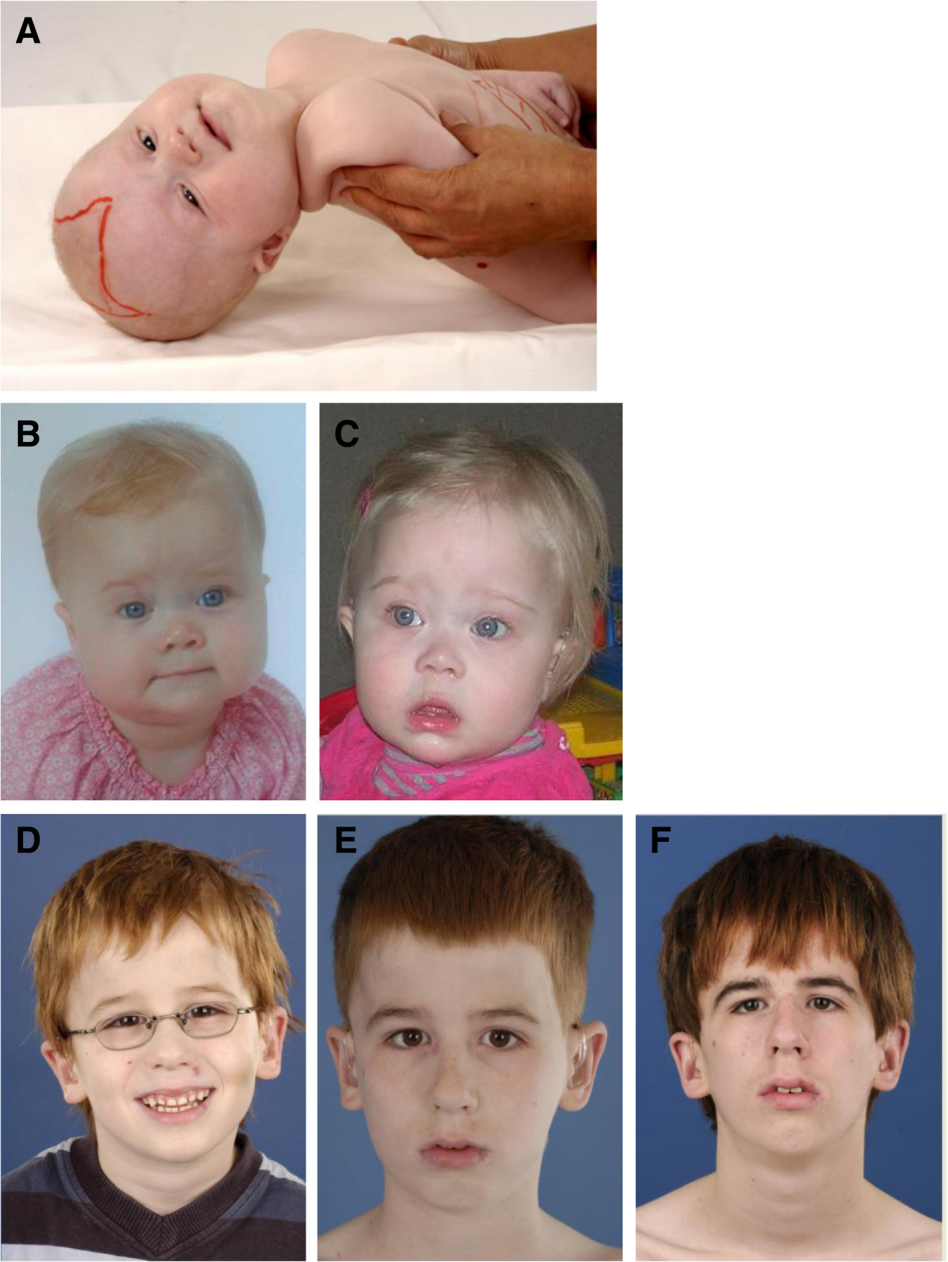
Craniofacial dysmorphic features in ZSD patients developing over time **a**. Photograph of a 6-month-old girl with typical craniofacial dysmorphia. Note the epicantal folds, high forehead, broad nasal bridge and hypoplastic supraorbital ridges. The anterior fontanel is drawn and enlarged. **b**-**c**. Girl with a ZSD at the age of 9 months (**b**) and at the age of 1 year and two months (**c**). Less pronounced facial dysmorphism is present: a high forehead is seen, a broad nasal bridge, hypoplastic supraorbital ridges, anteverted nares and more subtle epicantal folds. **d-f.** Photograph of a male with a ZSD at the age of 5 years (**d**), 10 years (**e**) and 15 years (**f**). No evident facial dysmorphic features can be recognized, although the ears seem to be slightly low-set. Written informed consent was obtained from the parents of all patients for publication of these images

ZSD patients within this group typically present in the neonatal period with hepatic dysfunction and profound hypotonia resulting in prolonged jaundice and feeding difficulties. Epileptic seizures are usually present in these patients. Characteristic dysmorphic features can usually be found, of which the facial dysmorphic signs are most evident (Fig. 2a). Sensorineural deafness and ocular abnormalities like retinopathy, cataracts and glaucoma are typical but not always recognized at first presentation. Brain magnetic resonance imaging (MRI) may show neocortical dysplasia (especially perisylvian polymicrogyria), generalized decrease in white matter volume, delayed myelination, bilaterial ventricular dilatation and germinolytic cysts [23]. Neonatal onset leukodystrophy is rarely described [25]. Calcific stippling (chondrodysplasia punctata) may be present, especially in the knees and hips. The neonatal-infantile presentation grossly resembles what was originally described as classic ZS. Prognosis is poor and survival is usually not beyond the first year of life.

### Childhood presentation

These patients show a more varied symptomatology than ZSD patients with a neonatal-infantile presentation. Presentation at the outpatient clinic usually involves delayed developmental milestone achievement. Ocular abnormalities comprise retinitis pigmentosa, cataract and glaucoma, often leading to early blindness and tunnel vision [26]. Sensorineural deafness is almost always present and usually discovered by auditory screening programs. Hepatomegaly and hepatic dysfunction with coagulopathy, elevated transaminases and (history of) hyperbilirubinemia are common. Some patients develop epileptic seizures. Craniofacial dysmorphic features are generally less pronounced than in the neonatal-infantile group (Fig. 2b, c). Renal calcium oxalate stones and adrenal insufficiency may develop. Early-onset progressive leukodystrophy may occur, leading to loss of acquired skills and milestones in some individuals. The progressive demyelination is diffuse and affects the cerebrum, midbrain and cerebellum with involvement of the hilus of the dentate nucleus and the peridentate white matter [23]. Sequential imaging in three ZSD patients showed that the earliest abnormalities related to demyelination were consistently seen in the hilus of the dentate nucleus and superior cerebellar peduncles, chronologically followed by the cerebellar white matter, brainstem tracts, parieto-occipital white matter, splenium of the corpus callosum and eventually involvement of the whole of the cerebral white matter [27]. The above described rapid progressive leukodystrophy, in combination with other symptoms described here, resemble what was originally described as NALD. A small subgroup of patients develop a relatively late-onset white matter disease, but no patients with late-onset rapid progressive white matter disease after the age of five have been reported [28]. Prognosis depends on what organ systems are primarily affected (i.e. liver) and the occurrence of progressive cerebral demyelination, but life expectancy is decreased and most patients die before adolescence.

### Adolescent-adult presentation

Symptoms in this group are less severe, and diagnosis can be in late child- or even adulthood [29]. Ocular abnormalities and a sensorineural hearing deficit are the most consistent symptoms. Craniofacial dysmorphic features can be present, but may also be completely absent (Fig. 2d-f). Developmental delay is highly variable and some patients may have normal intelligence. Daily functioning ranges from completely independent to 24 h care. It is important to emphasize that primary adrenal insufficiency is common and is probably under diagnosed [30]. In addition to some degree of developmental delay, other neurological abnormalities are usually also present: signs of peripheral neuropathy, cerebellar ataxia and pyramidal tract signs. The clinical course is usually slowly progressive, although the disease may remain stable for (many) years [31]. Slowly progressive, clinically silent leukoencephalopathy is common, but MRI may be normal in other cases [23].

## Etiology and pathophysiology

ZSDs are caused by mutations in one of the 13 different *PEX* genes. *PEX* genes encode proteins called peroxins and are involved in either peroxisome formation, peroxisomal protein import, or both. As a consequence, mutations in *PEX* genes cause a deficiency of functional peroxisomes. Cells from ZSD patients either entirely lack functional peroxisomes, or cells can show a reduced number of functional peroxisomes or a mosaic pattern (i.e. a mixed population of cells with functional peroxisomes and cells without) [1, 32, 33]. Peroxisomes are involved in many anabolic and catabolic metabolic processes, like biosynthesis of ether phospholipids and bile acids, α- and β-oxidation of fatty acids and the detoxification of glyoxylate and reactive oxygen species. Dysfunctional peroxisomes therefore cause biochemical abnormalities in tissues, but also in readily available materials like plasma and urine [3, 15] (summarized in Table 1). There is a reasonable genotype-phenotype correlation [24]. Approximately 60 % of ZSD patients have biallelic *PEX1* mutations and almost 90 different mutations in *PEX1* have been reported so far [34]. Detailed and up to date information about *PEX* gene mutations is available through the dbPEX gene database (http://www.dbpex.org).

**Table 1:** Peroxisome functions and their biochemical consequences and possible clinical relevance in ZSDs

Peroxisome function	Biochemical consequence	Possible clinical relevance
β-oxidation of VLCFA (≥C22)	Impaired chain shortening of VLCFA, last step in DHA synthesis is impaired	Brain, nerve and adrenal damage due to VLCFA tissue accumulation, DHA deficiency affects brain function and vision
β-oxidation of methyl-branched chain fatty acid, DHCA and THCA	Impaired chain shortening of DHCA, THCA and pristanic acid	Pristanic acid accumulation affects brain function, accumulation of DHCA and THCA causes liver toxicity and probably also brain damage
α-oxidation of fatty acids	Impaired (pre-) degradation of methyl branched phytanic acid	Retinal degeneration, brain and nerve damage due to phytanic acid accumulation
Fatty acid racemization	Reduced convertion of pristanoyl-CoA and C27-bile acyl-CoAs into stereoisomers before β-oxidation	Tissue accumulation of DHCA, THCA, pristanic- and phytanic acid
Ether phospholipid (plasmalogen) biosynthesis	Impaired formation of ether phospholipids	Plasmalogen deficiency gives rise to growth- and psychomotor retardation, cataract and bone development anomalies
Glyoxylate detoxification	Conversion of glyoxylate into oxalate, a toxic metabolite	Accumulation leads to calcium oxalate renal stones
L-lysine oxidation	Impaired L-pipecolic acid degradation	Accumulation of pipecolic acid, no clinical consequences known [78]
Hydrogen peroxide detoxification	Decreased catabolism of hydrogen peroxide	Increased reactive oxidant damage

## Diagnosis

**Fig. 3:**
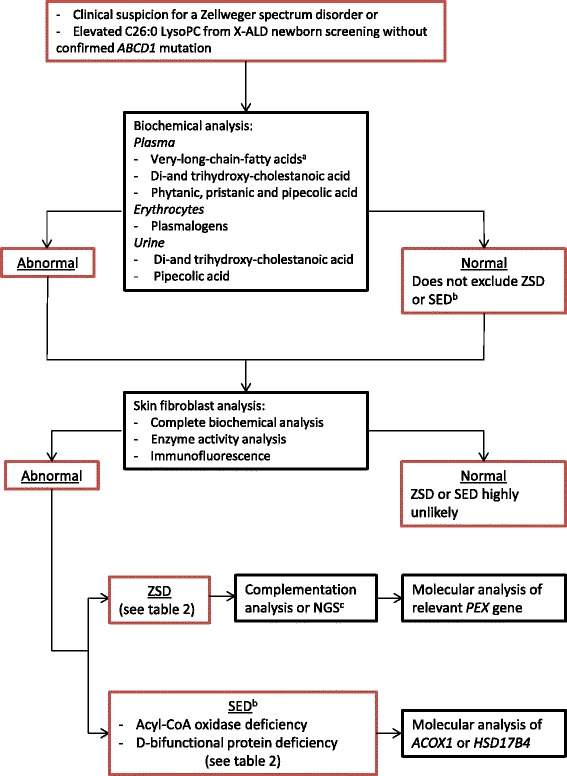
Diagnostic flow-chart for ZSDs. **a** Very long chain fatty acids: C26:0, C24:0/C22:0 ratio, C26:0/C22:0 ratio. **b** Single enzyme deficiency with phenotypical ZSD similarities like ACOX1 deficiency and DBP deficiency. **c** Next generation sequencing (NGS) of all *PEX* genes is advised when complementation analysis is not practicable

If a ZSD is clinically suspected the first step to confirm the diagnosis is by biochemical testing in readily accessible materials like blood and urine. This testing includes measurement of VLCFAs, the peroxisomal bile acid intermediates di- and trihydroxycholestanoic acid (DHCA, THCA), the branched-chain fatty acids phytanic and pristanic acid, and pipecolic acid in plasma, plasmalogen levels in erythrocytes, and C26:0-lysoPC in dried blood spots. Additionally, bile acids and oxalic acid can be analyzed in urine [24]. It is important to note that relatively mild ZSD patients may have (near) normal biochemical tests in plasma and urine [35–37]. If clinical suspicion of a ZSD is high and peroxisomal parameters in blood and urine are normal, further testing in fibroblasts is recommended, including culturing the fibroblasts at 40 °C [35]. Further fibroblast testing is also required to differentiate between ZSDs and certain peroxisomal single enzyme deficiencies, and to perform complementation studies to pinpoint the defective *PEX* gene. Subsequent mutation analysis of the defective *PEX* gene is done in all patients to confirm the diagnosis. A diagnostic flowchart is provided (Fig. 3). With increasing availability and reliability of next generation sequencing it is possible that genetic tests will become first tier tests in the future. However, biochemical testing in blood and/or fibroblasts is still required in these cases to confirm pathogenicity of the identified mutations and to characterize the extent of the deficiency.

## Differential diagnosis

Differential diagnosis varies with the age of presentation and most prominent symptoms at presentation (Table 2). In newborns, ZSDs with hypotonia are most often confused with other conditions presenting with profound hypotonia including chromosomal abnormalities. The most important differential disorders to consider when suspecting a ZSD is the group of single peroxisomal enzyme deficiencies. Especially Acyl-CoA oxidase type 1 (ACOX1) deficiency and D-bifunctional protein (DBP) deficiency show great overlap and in some cases, especially in the neonatal-infantile and childhood period, can be clinically indistinguishable from ZSDs [38, 39]. Also MRI-features in DBP-deficiency resemble those of ZSD patients [27]. Differentiation is possible with biochemical and genetic tests as summarized in Table 3. Dependent on the most prominent presenting symptom such as retinitis pigmentosa, cerebellar ataxia or adrenal insufficiency, other single peroxisomal enzyme deficiencies like classical Refsum disease, alpha-methylacyl-CoA racemase deficiency or X-ALD should be considered.

**Table 2:** Differential diagnosis of ZSDs based on the most prominent presenting symptom

Main presenting symptom	Differential diagnosis
Hypotonia in newborns	Chromosomal abnormalities (Down syndrome, Prader-Willi syndrome)
	Congenital infections (cytomegalovirus, rubella, herpes simplex, toxoplasmosis)
	Hypoxic ischemic encephalopathy
	Cerebral malformations
	Other metabolic disorders (acid maltase deficiency, carnitine deficiency, cytochrome-c-oxidase deficiency)
	Other peroxisomal disorders (acyl-CoA oxidase type 1 deficiency, D-bifunctional protein deficiency)
	Spinal muscular atrophy
	Congenital muscular dystrophies
	Congenital myopathies
	Hereditary motor and sensory neuropathy
Bilateral cataract	Idiopathic
	Congenital infections
	Other peroxisomal disorders (rhizomelic chondrodysplasia punctata, classical Refsum disease, 2-methylacyl-CoA racemase deficiency)
	Other metabolic disorders (galactosemia)
	Lowe syndrome
Sensorineural hearing loss with retinitis pigmentosa	Usher syndrome type I,II
	Other peroxisomal disorders (classical Refsum disease)
	Mitochondrial disorders
	Cockayne syndrome
	Alport syndrome
	Waardenburg syndrome
Adrenocorticol insufficiency	Autoimmune adrenalitis
	Infectious adrenalitis
	Adrenal hemorrhage
	Adrenal hypoplasia
	X-linked adrenoleukodystrophy
	Deficient cholesterol metabolism
	Familial glucocorticoid deficiency

**Table 3:** Differences in biochemical characteristics of ZSDs and phenotypical similar single enzyme deficiencies

	ZSD	DBP-D	ACOX1-D	Remarks
Plasma				
Very long chain fatty acids^a^	↑^b^	↑^b^	↑^b^	False positives possible in ketogenic diets, hemolyzed samples and peanut rich diet.
Di- and trihydroxycholestanoic acid	↑^b^	N-↑	N	
Phytanic acid	N-↑	N-↑	N	Derived from dietary sources only; dependent on dietary intake. Normal in newborns.
Pristanic acid	N-↑	N-↑	N	Derived from dietary sources only (direct and indirectly via phytanic acid). Normal in newborns.
Erythrocytes				
Plasmalogen level	↓-N	N	N	
Blood spot				
C26:0 lysophosphatidylcholine	↑	↑	↑	
Fibroblasts				
Plasmalogen synthesis	↓	N	N	
DHAPAT	↓	N	N	
Alkyl DHAP synthase	↓	N	N	
C26:0 β-oxidation	↓	↓	↓	
Pristanic acid β-oxidation	↓	↓	N	
Acyl-CoA oxidase 1	↓-N	N	↓	
D-Bifunctional protein	↓-N	↓	N	
Phytanic acid α-oxidation	↓	N	N	
Phytanoyl CoA hydroxylase	↓	N	N	
Peroxisomes	↓	N	N	Peroxisomal mosaicism can be present in ZSD. In DBP- and ACOX1-deficiency abnormal peroxisomal morphology may be present.
Mutant gene	*PEX*1,2,3,5,6,10,11β,12,13,14,16,19,26	*HSD17B4*	*ACOX*	

^a^Very long chain fatty acids: C26:0, C24:0/C22:0 ratio, C26:0/C22:0 ratio

^b^May be minimally abnormal to normal in exceptional cases

## Genetic counseling and antenatal diagnosis

Because of the poor outcome and high disease burden associated with the majority of ZSDs, genetic counseling should be offered to parents of affected children. Carriers can be offered prenatal- or preimplantation genetic diagnosis. Before prenatal genetic testing can be performed the familial pathogenic mutation (s) in one of the *PEX* genes need (s) to be identified [1]. If the *PEX* mutations are unknown or cannot be detected, biochemical prenatal testing for ZSD is possible in chorionic villus biopsy material, cultured chorionic villus cells or cultured amniocytes. Biochemical prenatal testing can only be performed in case of clear biochemical abnormalities in cells from the index patient [15].

## Clinical management and treatment

Because no curative therapy for patients with a ZSD exists, intervention is supportive and based on symptoms. Past- and current supportive therapeutic options are summarized in Table 4.

**Table 4:** Supportive therapeutic options in ZSDs

Symptom/disease	Treatment/intervention
Adrenal insufficiency	Cortisone
Coagulopathy	Vitamin K suppletion
Enamel hypoplasia	Dentist referral
Epilepsy	Standard antiepileptic drugs
Hearing impairment	Hearing aids, cochlear implant
High phytanic acid plasma level	Phytanic acid restricted diet
Hyperoxaluria	Oral citrate treatment Sufficient fluid intake
Insufficient calory intake	Gastrostomy
Low levels of fat-soluble vitamins (A, D, E)	Vitamin suppletion
Visual impairment	Cataract removal, glasses and ophthalmologist referral

### Docosahexaenoic acid

Docosahexaenoic acid (DHA; C22:6ω3) is a long-chain polyunsaturated fatty acid important for retinal and brain function [40, 41]. Tetracosahexaenoic acid (C24:6ω3) undergoes one cycle of peroxisomal beta-oxidation to be converted to DHA [4], leading to reduced levels of DHA when peroxisomes are absent. Because ZSD patients often have low levels of DHA in membranes of erythrocytes, supplementation of DHA was suggested to be a possible therapy. Although some studies have claimed a beneficial effect of DHA supplementation [42, 43], a randomized double-blind placebo controlled trial showed that DHA treatment leads to increased DHA levels in plasma, but no improvement of visual function and growth could be observed [44].

### Lorenzo’s oil

Lorenzo’s oil (i.e. 4:1 mix of glyceryl trioleate and glyceryl trierucate) therapy was originally developed for the single peroxisomal enzyme deficiency X-ALD, and was shown to lower VLCFAs in plasma [45], but had no effect on disease progression [46, 47]. Some studies reported lowering of the VLCFA levels in plasma by Lorenzo’s oil in ZS babies [48, 49]. However, based on data of studies in X-ALD individuals, there is no reason to expect that Lorenzo’s oil will be beneficial for ZSD patients at this point.

### Cholic acid

Cholic acid is a primary C24 bile acid, involved in for instance the absorption of fat-soluble vitamins. Cholic acid is formed from its precursor THCA by one peroxisomal beta-oxidation cycle. The peroxisomal C27-bile acid intermediates DHCA and THCA accumulate in ZSDs and are considered to be more toxic than the primary C24 bile acids due to their altered physical properties and are believed to contribute to the liver disease in ZSDs (e.g. dysfunction and liver fibrosis) [50]. The bile acid intermediates are only partly conjugated and are less well excreted than C24 bile acids contributing to cholestasis. We hypothesize that DHCA and THCA cross the blood–brain barrier and cause central nerve system damage. Several case reports have described a beneficial effect of cholic acid in ZS babies, supported by reduced urinary and plasma excretion of DHCA/THCA [51, 52]. Clinically there was increased growth and an increase in the levels of fat-soluble vitamins. Furthermore, bile acid treatment in mice was shown to improve hepatic disease [53]. Limitations of the studies so far, however, are the small number of treated patients and short follow-up. Current evidence is insufficient to conclude that cholic acid treatment is beneficial for patients with a ZSD. The Food and Drug Administration recently approved cholic acid as a safe treatment for ZSD patients in the United States. However, efficiency should be demonstrated in large clinical trials before this treatment can be implemented.

### Plasmalogen precursors

Due to a deficiency of the first peroxisomal steps in the biosynthesis of plasmalogens [54], ZSD patients may have low levels of plasmalogens. Plasmalogens play a critical role in cell membranes and as anti-oxidants [55]. It was suggested that supplementation with precursors of plasmalogens (batyl alcohol) could be beneficial for ZSD patients, as import of these alkylglycerols proceeds normally. Several case reports have described an increase in erythrocyte plasmalogen levels after treatment and improvement of clinical symptoms in some patients [56–58]. Although never studied systematically, ether lipid therapy could be of interest for ZSD.

### Citrate

The toxic metabolite oxalate accumulates in plasma and urine from ZSD patients [4]. This causes renal calcium oxalate stones. In a large cohort of Dutch ZSD patients a high prevalence of 83 % of renal calcium oxalate stones was shown [59]. For this reason, patients should be screened for the presence of high levels of oxalic acid in urine yearly. To prevent the formation of renal stones, patients with hyperoxaluria should start oral citrate treatment. Furthermore, sufficient fluid intake is recommended [60].

### Supportive care

All ZSD patients need to be screened for adrenal insufficiency [30], epilepsy, low levels of fat-soluble vitamins, (partly) vitamin K dependent coagulopathy, high levels of phytanic acid, hearing or visual impairment and enamel hypoplasia. They should be treated according to the identified abnormalities, e.g. supplementation of cortisone, anti-epileptic drugs, vitamins and/or a phytanic restricted diet. Because supplementation of cortisone is associated with severe side effects, such as growth suppression and osteoporosis [61], only patients with a true insufficiency (i.e. altered Synacthen test) should be treated. A phytanic acid restricted diet is only necessary when levels of phytanic acid are extremely high and is not recommended when levels are moderately increased, as sufficient intake of calories is more decisive. Hearing and visual impairment should be (partly) corrected by hearing aids and glasses, with ophthalmologic and audiological evaluations yearly. Enamel hypoplasia, present in nearly all patients, should be followed-up by a dentist [62, 63]. Some patients will need a gastrostomy to provide adequate intake of calories.

### Current/future developments

Several compounds that stimulate peroxisomal biogenesis and function *in vitro* [64–66] were discovered recently and clinical trials are ongoing (clinicaltrails.gov: NCT01838941). Hopefully, some of these compounds will be able to rescue or improve peroxisomal function in patients. The greatest beneficial effect is expected in patients whose fibroblasts showed a temperature sensitivity with worsening of the phenotype when cultured at 40 °C and improvement of peroxisomal functions at 30 °C [67, 68]. In addition to these new compounds, the effect of cholic acid is currently under investigation (controlled-trials.com: ISRCTN96480891) in a large cohort of ZSD patients.

Although never tested in ZSD patients, gene therapy with or without tissue specific targeting might be a potential treatment. Several years ago gene therapy was already proposed for X-ALD [69]. Although promising, gene therapy still needs to be optimized to be feasible for patients [70]. First, studies have to be conducted in the recently published mild *PEX1* mouse model [71], before a human trial can be initiated.

An orthotopic liver transplantation was described in a single 6-month old ZSD patient and hepatocytes transplantation in another 4-year old patient [72, 73]. It resulted in decreased concentrations of VLCFAs and pipecolic acid, and improved bile acid profiles. However, the effect on long-term disease course has not been reported.

Although bone marrow transplantation (BMT) is an established therapy for the cerebral childhood form of X-ALD [74], there are no reports describing BMT in ZSD patients. BMT would be of interest for those patients who develop leukodystrophy in infancy. However, with the current knowledge it is impossible to predict if patients will develop this rapid progressive leukodystrophy. Recently, a retrospective study revealed that patients with X-ALD still develop an adrenomyelopathy phenotype after BMT [75]. Nevertheless, BMT could possibly be beneficial for a subgroup of patients within the ZSD spectrum, but first new techniques/markers that can predict whether or not patients will develop a severe progressive leukodystrophy have to be elucidated.

## Prognosis

Although a rough genotype-phenotype correlation exists for several *PEX* genes, such as *PEX1* and *PEX26* [76, 77], the severity and progression of the disease is difficult to predict for individual patients. This will become more relevant as newborn screening is implemented. As a consequence of newborn screening for X-ALD by C26:0-lysoPC in several countries ZSD will also be diagnosed at birth. Children with the severe phenotype (neonatal-infantile presentation with severe clinical symptoms) have a poor prognosis and these patients usually die within the first year of life. Patients that present in childhood or adolescence usually have a better prognosis, but can develop progressive liver disease or leukodystrophy and deteriorate. If progressive liver disease or leukodystrophy occurs prognosis is poor. The remaining milder individuals can reach adulthood without progression or with long periods of stabilization. When progression occurs, it is mainly related to peripheral neuropathy and pyramidal signs, while cognition remains stable [31].

## Unresolved questions

The effect of cholic acid is based upon case reports only, but within the coming years the clinical effects will be investigated in larger cohorts. In addition, results of ongoing trials will be published. An important limitation to consider when interpreting the data of these trials is the broad spectrum of severe and milder clinical phenotypes and associated biochemical variations within these cohorts. Furthermore, the natural course of the disease can lead to false conclusions, as peroxisomal metabolites were shown to fluctuate and decline with age [31]. A large prospective natural history study is therefore needed. We and others, recently started collecting the data of a large prospective cohort of ZSD patients (clinicaltrails.gov: NCT01668186).

Second, plasma levels of peroxisomal metabolites do not correlate well with disease severity, as they generally decrease with age. Furthermore, therapies like DHA and Lorenzo’s oil improved plasma levels of DHA and C26:0, albeit no effect on the clinical phenotype has been observed. This is possibly related to differences in expression or activity of peroxisomes in the targeted tissue. Therefore, using plasma levels as a surrogate outcome in clinical trials is not recommended. New biochemical outcome parameters that correlate with disease progression are necessary, such as analysis of markers for peroxisomal dysfunctions in lymphocytes.

The pathophysiology of ZSD is still poorly understood. Similar to the cerebral form of X-ALD, it is still not clear when or why ZSD patients develop severe rapid progressive leukodystrophy. The recently constructed mild *PEX1* mouse model [71] and natural history studies will help to answer these questions.

## Conclusions

Because of the recently implemented newborn screening, more medical doctors in different specialties (e.g. pediatricians, clinical geneticist and neurologists) will encounter patients with a ZSD. ZSDs are clinically heterogeneous with high morbidity in almost all patient and mortality in some. Although treatment is currently only symptomatic, it is important to initiate proper supportive therapy to improve quality of life of these patients.

## Abbreviations

ACOX1: acyl-CoA oxidase type 1
BMT: bone marrow transplant
C26:0-lysoPC: C26:0-lysophosphatidylcholine
DBP: d-bifunctional protein
DHA: docosahexanoic acid
DHCA: dihydroxycholestanoic acid
IRD: infantile Refsum disease
MRI: magnetic resonance imaging
NALD: neonatal adrenoleukodystrophy
THCA: trihydroxycholestanoic acid
VLCFAs: very long chain fatty acids
X-ALD: x-linked adrenoleukodystrophy
ZS: Zellweger syndrome
ZSD: Zellweger spectrum disorder
